# Platelet-Derived Growth Factor-BB Inhibits Intervertebral Disc Degeneration via Suppressing Pyroptosis and Activating the MAPK Signaling Pathway

**DOI:** 10.3389/fphar.2021.799130

**Published:** 2022-01-14

**Authors:** Weikang Zhang, Yuhang Gong, Xiaohang Zheng, Jianxin Qiu, Ting Jiang, Lihua Chen, Fangying Lu, Xinhui Wu, Fengmin Cheng, Zhenghua Hong

**Affiliations:** ^1^ Orthopedic Department, Taizhou Hospital Affiliated to Wenzhou Medical University, Linhai, China; ^2^ Enze Medical Research Center, Taizhou Hospital Affiliated to Wenzhou Medical University, Linhai, China

**Keywords:** degeneration, pyroptosis, extracellular matrix, intervertebral disc, PDGF-BB

## Abstract

Platelet-derived growth factor-BB (PDGF-BB) is a cytokine involved in tissue repair and tumor progression. It has been found to have expression differences between normal and degenerative intervertebral discs. However, it is not clear whether PDGF-BB has a protective effect on intervertebral disc degeneration (IDD). In this experiment, we treated nucleus pulposus cells (NPCs) with IL-1β to simulate an inflammatory environment and found that the extracellular matrix (ECM) anabolic function of NPCs in an inflammatory state was inhibited. Moreover, the induction of IL-1β also enhanced the expression of NLRP3 and the cleavage of caspase-1 and IL-1β, which activated the pyroptosis of NPCs. In this study, we studied the effect of PDGF-BB on IL-1β-treated NPCs and found that PDGF-BB not only significantly promotes the ECM anabolism of NPCs, but also inhibits the occurrence of pyroptosis and the production of pyroptosis products of NPCs. Consistent with this, when we used imatinib to block the PDGF-BB receptor, the above-mentioned protective effect disappeared. In addition, we found that PDGF-BB can also promote the ECM anabolism of NPCs by regulating the ERK, JNK, PI3K/AKT signaling pathways, but not the P38 signaling pathway. *In vivo* studies, mice that blocked PDGF-BB receptors showed more severe histological manifestations of intervertebral disc degeneration. In summary, our results indicate that PDGF-BB participates in inhibiting the occurrence and development of IDD by inhibiting pyroptosis and regulating the MAPK signaling pathway.

## Introduction

Intervertebral disc degeneration (IDD) is recognized as the main cause of low back pain (Wuertz and Haglund, 2013; Risbud and Shapiro, 2014; Yelin et al., 2016; Kos et al., 2019). As the world’s population ages, it will further increase the social burden (Vergroesen et al., 2015). So far, several major theories have been proposed to explain the causes of IDD, including inflammation, apoptosis, aging, and biomechanical burden (Stokes and Iatridis, 2004; Le Maitre et al., 2007; Ding et al., 2012; Liu et al., 2018), but the exact cause of IDD remains to be clarified (Feng et al., 2016).

Under normal circumstances, the anabolism and catabolism of the extracellular matrix (ECM) of the intervertebral disc (IVD) are kept in balance (Cui and Zhang, 2020; Chen et al., 2021). ECM is the main component of the intervertebral disc. It is produced by cells and constitutes the microenvironment for cell survival, and it is mainly responsible for maintaining the structure and function of the intervertebral disc (Guo et al., 2021; Zhao et al., 2021). When the intervertebral disc is in an inflammatory environment or infection, it is often accompanied by a decrease in ECM anabolism and an increase in catabolism (Wang et al., 2016; Li et al., 2017; Tang et al., 2021). This imbalance will lead to changes in the water content, height reduction, and abnormal stress distribution in the intervertebral disc, thereby accelerating the degeneration of the intervertebral disc (Lan et al., 2021). Therefore, promoting the synthesis of extracellular matrix of the intervertebral disc is considered to be one of the strategies for the treatment of IDD.

Platelet-derived growth factor-BB (PDGF-BB) is a peptide regulator that stimulates tissue cell growth. It is activated and released by disintegrated platelets when blood coagulates. In addition, macrophages, endothelial cells and epithelial cells can also synthesize and release PDGF. More and more attention has been paid to the regulation of growth factors on the production of ECM and cell proliferation of intervertebral discs. However, the current research on PDGF-BB mainly focuses on tumor and angiogenesis. Although studies have shown that PDGF-BB can inhibit the apoptosis of nucleus pulposus cells (NPCs) induced by interleukin-1β (IL-1β), its effect on the extracellular matrix and the specific regulatory mechanism are still unclear (Gruber et al., 2000; Paglia et al., 2016). Therefore, in-depth exploration of the regulation mechanism of PDGF-BB on the intervertebral disc is helpful to provide reliable experimental basis and theoretical support for the treatment of degenerative disc disease.

Pyroptosis, also known as cell inflammatory necrosis, is a kind of programmed cell death, which is manifested by the continuous expansion of cells until the cell membrane ruptures, which leads to the release of cell contents and activates a strong inflammatory response (Robinson et al., 2002; Zhou et al., 2021). Apoptosis was originally thought to be the main form of death of nucleus pulposus cells, but recent studies have shown that nucleus pulposus cells in an inflammatory environment will die in a more dangerous form of pyroptosis than apoptosis (Li et al., 2014; Haneklaus and O’Neill, 2015; Man et al., 2017). Pyroptosis cells release large amount of metabolites such as interleukin-1β and interleukin-18, which will further trigger a strong inflammatory response. The MAPK pathway plays an important role in regulating the balance of anabolism and catabolism of the intervertebral disc (Dai et al., 2021; Zhang et al., 2021b). This pathway can be activated by inflammatory factors, growth factors, pressure changes, and other factors. Some scholars have discovered that PDGF-BB can regulate the function of nucleus pulposus cells by activating the ERK pathway (Pratsinis and Kletsas, 2015). To this end, we studied the effects of PDGF-BB on three MAPK signaling pathways.

## Materials and Methods

### Clinical Tissue Samples

All specimens were taken from patients undergoing spinal surgery in the Department of Orthopedics, Taizhou Hospital Affiliated to Wenzhou Medical University. Among them, the relatively normal nucleus pulposus tissue was taken from five young patients who underwent surgical treatment for spinal fractures, and all five patients were graded I to II according to the Pfirrmann grading standard. The degenerated nucleus pulposus tissue was taken from five middle-aged and elderly patients who underwent surgical treatment for degenerative disc diseases, and their Pfirrmann grades were all gardes IV–V. This study was approved by the Ethics Committee of Taizhou Hospital in Zhejiang Province (Approval number: K20210206), and written informed consent was obtained from all participants. Basic information of the patient is listed in Table 1.

**TABLE 1:** Basic information of the patient.

Patient	Gender	Age	Reason for the operation	Lesion	Pfirrmann grade
1	Male	66	Herniated disc	L4/L5	V
2	Female	70	Herniated disc	L5/S1	IV
3	Male	63	Herniated disc	L4/L5	V
4	Female	85	Herniated disc	L4/L5	IV
5	Female	65	Herniated disc	L5/S1	V
6	Female	35	Vertebral fracture	T12	I
7	Male	19	Vertebral fracture	C4-C6	I
8	Male	30	Vertebral fracture	L4-L5	II
9	Male	49	Vertebral fracture	L2	II
10	Male	16	Vertebral fracture	C5	I

### Drugs and Reagents

RHu PDGF-BB, IL-1β and imatinib mesylate were purchased from MedChemExpress, United States (cat. nos. HY-P7055; HY-P7097; HY-50946); PDGF-BB polyclonal antibody, collagen II monoclonal antibody and aggrecan neo polyclonal antibody were purchased from Thermo Fisher Scientific, United Kingdom (cat. nos. PA5-88272; MA5-12789; PA1-1746); MMP3 antibody, MMP9 antibody, Adamts4 antibody, Adamts5 antibody and Caspase1 antibody were purchased from Abcam, United Kingdom (cat. nos. ab52915; ab76003; ab185722; ab41037; ab179515); PDGFR antibody, P-PDGFR antibody, P-P38 antibody and P-JNK antibody were purchased from Hangzhou Huaan Biotechnology Co., Ltd., China (cat. nos. SN0646; R1510-44; ER2001-52; ET1601-28); P-PI3K antibody, PI3K antibody, P-AKT antibody, AKT antibody, P38 antibody, JNK antibody, P-ERK antibody, ERK antibody, β-actin antibody and NLRP3 antibody were purchased from Cell Signaling Technology, United States (cat. nos.17366; 4292; 9271; 4691; 9212S; 9252S; 4370; 4695; 4970; ab263899); IL-1β antibody was purchased from ABclonal Technology Co., Ltd., China (cat. no. A1112).

### High-Density Cell Culture

Rat nucleus pulposus cells were donated by Professor Di Chen from Rush University in the United States. The culture conditions were 94% DMEM medium, 5% fetal bovine serum, and 1% penicillin-streptomycin double antibody. Cells are cultured statically at 37°C and 5% CO_2_, and the medium is changed every other day. In all experiments, the concentration of dimethyl sulfoxide was less than 1:1,000.

For High-density culture, 10 µl of nucleus pulposus cells with a density of 10^7^ cells/ml were seeded in a 24-well plate for 6 h. After the cells are fully attached, the nucleus pulposus cells culture medium and corresponding drugs are added, and the culture medium is changed every other day and incubated for 7 days. The cells were then fixed with paraformaldehyde and stained according to the instructions of the toluidine blue staining kit. Finally, use EPSON V600 photo scanner (Japan) to scan and take pictures of the 24-well plate.

### Cell Viability Assay

Use CCK-8 cell proliferation/cytotoxicity test kit to detect the viability of nucleus pulposus cells treated with imatinib. Before using the kit, nucleus pulposus cells were seeded at a density of 2 × 10^3^ cells/well for 24 h and treated with a serial dilution of imatinib (from 0.078 to 40 nM) for 24, 48 and 72 h. At the specified time, aspirate the supernatant from each well, then add 100 μl of DMEM containing CCK-8 solution to each well according to the kit instructions and incubate at 37°C for 90 min. Finally, a Multiskan FC microplate photometer (Thermo Fisher Scientific, Waltham, MA) was used to measure the absorbance of each well.

### Immunofluorescence

NP cells were treated with IL-1β (10 ng/ml) and PDGF-BB (50 ng/ml) for 48 h. After fixing with 4% paraformaldehyde (PFA) for 15 min, it was permeabilized with 0.5% TritonX-100 for 30 min. Then block with 1% bovine serum albumin (BSA) for 30 min, and incubate overnight with anti-COL2 antibody (1:100) or anti-NLRP3 antibody (1:100). Incubate with fluorescently labeled secondary antibody for 30 min the next day. Finally, NP cells were counter-stained with DAPI for 10 min, observed and photographed under an immunofluorescence microscope.

### Flow Cytometry

The NP cells were digested by trypsin, collected, and fixed with 70% ethanol at 4°C for 1 h. Aspirate ethanol after centrifugation, add PBS solution and centrifuge again. Then stain with propidium iodide stain including RNase A for 30 min in the dark according to the manufacturer’s instructions (Beyotime, Shanghai, China). Finally, the apoptosis and cell cycle were evaluated by flow cytometry. Calculate the percentage of cells in G1, S, G2 and apoptotic cells by CytExpert software.

### Western Blot

The total protein of nucleus pulposus cells was lysed and extracted using radioimmunoprecipitation buffer (RIPA) (Biosharp, China) (100:1) containing phenylmethanesulfonyl fluoride (PMSF). The protein sample was centrifuged at 4°C, 12,000 rpm for 15 min, and then the protein concentration was determined using the BCA protein detection kit (AMEKO, Shanghai, China). Prepare the gel according to the instructions of the PAGE Gel Fast Preparation Kit (Epizyme, Shanghai, China), separate the protein samples, and then transfer them to the PVDF membrane (Bio-Rad, Hercules, CA, United States). And use blocking buffer (Biosharp, Hefei, China) to seal at room temperature for 50 min. Incubate the PVDF membrane with the primary antibody overnight at 4°C, and then incubate with the secondary antibody for 1 h at room temperature. Ultra-sensitive ECL chemiluminescence kit (NCM Biotech, Suzhou, China) and ImageQuant LAS 500 (GE Health Care, Fairfield, CT, United States) were used to detect antibody reactivity. ImageJ software quantitatively analyzes the band intensity.

### RNA Extraction and Quantitative PCR

The nucleus pulposus cells were seeded in a six-well plate for 24 h at a density of 5 × 10^5^ cells per well. Then, it was treated with a complete medium containing the drug or recombinant protein for 48 h. According to the manufacturer’s instructions, total RNA was extracted with TRIzol reagent (Gibco, United States). Then, the total RNA was reverse transcribed into cDNA using HiFiScript cDNA Synthesis kit (Cwbiotech, China) according to the manufacturer’s instructions. ABI 7300 plus real-time PCR system (Applied Biosystems, Foster City, CA, United States) and ChamQ Universal SYBR qPCR Master Mix (Vazyme, China) were used for relative quantitative real-time PCR analysis. Relavent primer sequences are listed in Table 2.

**TABLE 2:** The primers sequences for Real-time PCR.

Gene	Primer sequences (5′-3′)	
PDGF-BB	Forward	CAA​AGG​GAA​GCA​CCG​AAA​GTT
	Reverse	TTA​AAT​AAC​CCT​GCC​CAC​ACT​CTC
PDGF-BB receptor	Forward	ACCACGGCGATGAGAA
	Reverse	AGAGGGCGTCGGATAA
CyclinD1	Forward	GGAATTGATGCGTGATGT
	Reverse	ACCAGGTGCTGTGGAGTA
CDK4	Forward	ATG​GCT​GCC​ACT​CGA​TAT​GAA
	Reverse	TGC​TCC​TCC​ATT​AGG​AAC​TCT​C
Aggrecan	Forward	GAC​CAG​GAG​CAA​TGT​GAG​GAG
	Reverse	CTCGCGGTCGGGAAAGT
COL2	Forward	GAA​CAA​CCA​GAT​CGA​GAG​CA
	Reverse	CTC​TCC​AAA​CCA​GAT​GTG​CT
Adamts4	Forward	CGC​TGA​GTA​GAT​TCG​TGG​AGA​C
	Reverse	AGT​TGA​CAG​GGT​TTC​GGA​TGC
Adamts5	Forward	CGA​CAA​GAG​TCT​GGA​GGT​GAG
	Reverse	CGT​GAG​CCA​CAG​TGA​AAG​C
MMP3	Forward	GAC​CAG​GGA​CCA​ATG​GAG​ATG
	Reverse	TGA​GCA​GCA​ACC​AGG​AAT​AGG
MMP9	Forward	CCT​ACT​GCT​GGT​CCT​TCT​G
	Reverse	GGCTTCCTCCGTGATTCG
GAPDH	Forward	ATG​GGA​AGC​TGG​TCA​TCA​AC
	Reverse	GTG​GTT​CAC​ACC​CAT​CAC​AA

### Enzyme-Linked Immunosorbent Assay

The nucleus pulposus cells were treated with IL-1β and PDGF-BB for 48 h and then centrifuged at 4°C and 12,000 rpm for 10 min, and the supernatant was collected. Then use the specific rat IL-1β ELISA kit (Kenuodi, Hangzhou, China) and the specific rat IL-18 ELISA kit (Kenuodi, Hangzhou, China) to determine the content of the apoptotic product according to the instructions of the kit.

### *In Vivo* Animal Experiment

Animal experiments were approved by the Animal Ethics Committee of Taizhou Hospital (Approval number: TZY2021008). All animals were purchased from Shanghai Slack Laboratory Animal Co., Ltd. To evaluate the effect of imatinib on the intervertebral disc of mice, nine 12-week-old male C57BL/J6 mice were randomly divided into three groups (*n* = 3 per group): sham (with PBS injection), low dose (with 25 mg/kg imatinib) and high dose (with 50 mg/kg imatinib). Mice were intraperitoneally injected with a specific concentration of imatinib or an equivalent amount of PBS twice a week. All mice were euthanized after 8 weeks of intraperitoneal injection. Then, the intervertebral disc was collected and fixed in 4% paraformaldehyde for paraffin embedding.

For mouse intervertebral discs, we used the mouse intervertebral disc scoring system proposed by Tam et al. (2018) to assess the degeneration of the intervertebral disc.

### *In Vitro* Organoid Culture

After the 8-week-old mice were euthanized, the intervertebral disc and its adjacent vertebral bodies were separated under aseptic operation. The intervertebral disc was inoculated on a 12-well plate and cultured in organoid culture medium with or without IL-1β and imatinib for 96 h. Finally, the organoids were harvested and fixed in 4% paraformaldehyde for the next histological analysis.

### Histopathological Analysis and Immunohistochemical Examination

The specimens were fixed in formaldehyde, decalcified for 14 days, embedded in paraffin, and sectioned at 5 μm. IVD sections were stained with hematoxylin-eosin and Safranin O-Fast Green stains. The quantification of histological score is based on a new scoring system specifically for mouse IVD histomorphology, and the H-E image is used to quantify the disc height index.

For immunohistochemistry, the sections were deparaffinized, dehydrated, and incubated with 3% hydrogen peroxide for 10 min. After the sections were blocked with 1% goat serum albumin, the primary antibody was incubated overnight at 4°C. Then, the sections were incubated with HRP-conjugated secondary antibody for 1 h at room temperature. Finally, the sections were counterstained with hematoxylin. Image analysis of the NP areas stained in brown was carried out using ImageJ for quantification of the positive area.

### Statistical Analysis

All experiments were performed independently at least 3 times. Quantitative results are expressed as mean ± standard deviation (SD). The data were analysed via GraphPad Prism (United States). *p* value <0.05 were considered statistically significant.

## Results

### The Expression of Platelet-Derived Growth Factor-BB in Normal and Degenerative Intervertebral Disc is Different

**FIGURE 1:**
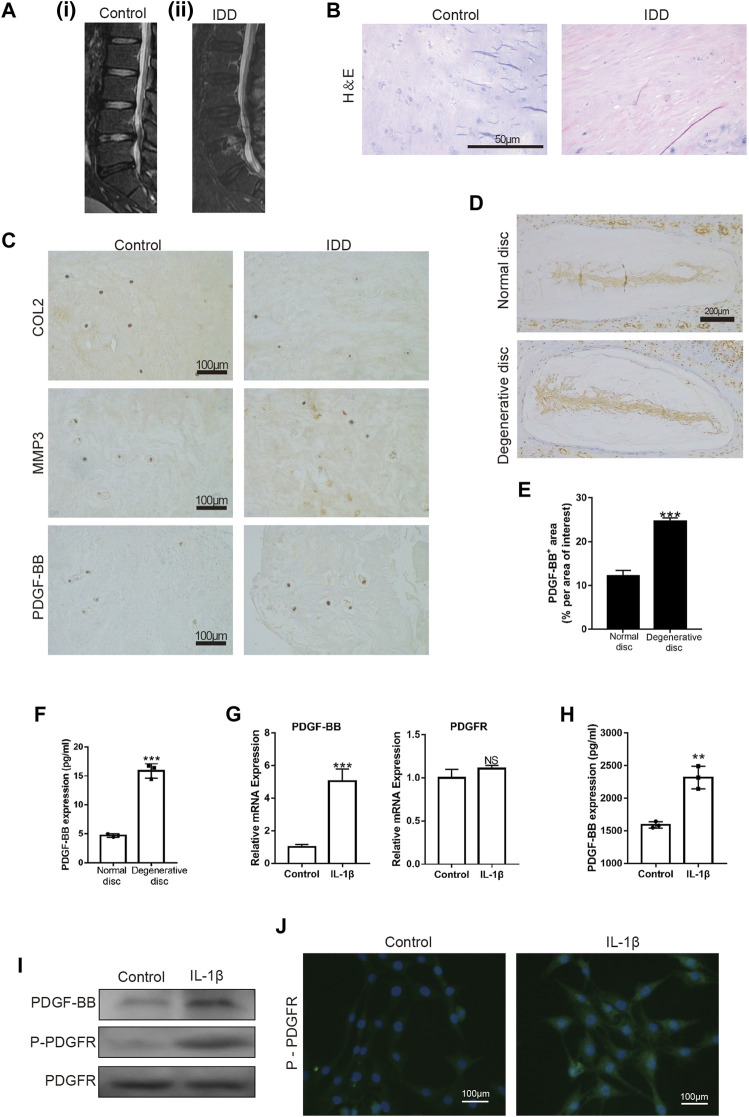
PDGF-BB is expressed in nucleus pulposus tissue and NP cells. **(A)** MRI of intervertebral discs; **(i)** patient with L4 vertebral fractures; **(ii)** patient with L4/5 disc herniation. **(B)** Hematoxylin and eosin staining of nucleus pulposus tissue in patients with vertebral body fracture and lumbar disc herniation. Scale bar, 50 μm. **(C)** Immunohistochemical images of COL2, MMP3 and PDGF-BB of human nucleus pulposus tissue. Scale bar, 100 μm. **(D,E)** Immunohistochemical images of PDGF-BB of mouse intervertebral disc. Average percentage of positive staining area of PDGF-BB. Scale bar, 200 μm. **(F)** ELISA shows the expression of PDGF-BB in mouse intervertebral disc. **(G–I)** Quantitative PCR, ELISA and Western blot were used to detect PDGF-BB of NP cells. **(J)** Immunofluorescence images of P-PDGFR of NP cells. Scale bar, 100 μm. All NP cells were treated with or without IL-1β (10 ng/ml) for 48 h. Data are presented as the mean ± SD. Significant differences between groups are indicated as ****p < 0.0001, ***p < 0.001, **p < 0.01.

First, we examined the intervertebral disc of different patients by MRI. The intervertebral disc segment in patients with lumbar intervertebral disc herniation (LDH) showed low signals on the MRI T2 (Figure 1A). H-E staining showed that the number of intervertebral disc cells in patients with lumbar intervertebral disc herniation was significantly less than that in patients with vertebral body fractures (Figure 1B). At the same time, we also detected the expression of COL2, MMP3 and PDGF-BB in the nucleus pulposus tissue. The immunohistochemical results showed that the expression of COL2 was decreased in LDH patients, while the expression of MMP3 was increased (Figure 1C). This suggests that the degeneration of the nucleus pulposus in patients with LDH is more serious than that in patients with vertebral fractures. It is worth noting that the expression of PDGF-BB in the nucleus pulposus of LDH patients was more than that of patients with vertebral fractures. The results of immunohistochemistry and ELISA showed that the expression of PDGF-BB in the degenerated intervertebral discs of mice is also increased (Figures 1D–F).

To verify whether PDGF-BB exists in NP cells, we detected the expression of PDGF-BB and PDGF receptor in rat nucleus pulposus cells by Western blot, qPCR and ELISA. Interestingly, the results showed that the treatment of IL-1β promotes the expression of PDGF-BB in NP cells (Figures 1G–I). In addition, immunofluorescence images suggested that IL-1β treatment promoted the phosphorylation of PDGF receptors on NP cells (Figure 1J). The above results suggest that PDGF-BB may be related to the degeneration of the intervertebral disc.

### Platelet-Derived Growth Factor-BB Inhibits the Degeneration of Organoids Cultured *In Vitro*

**FIGURE 2:**
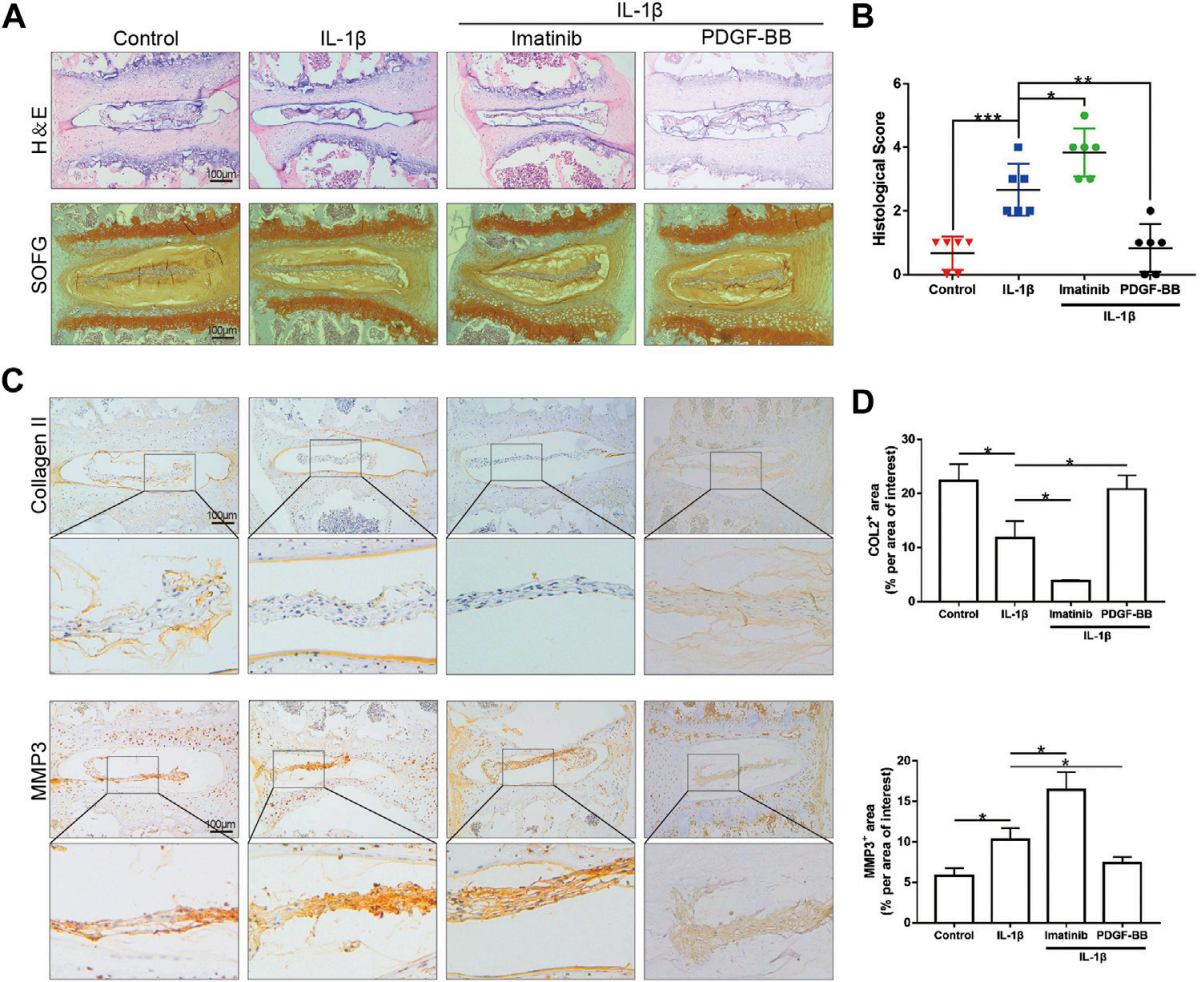
PDGF-BB inhibits the degeneration of organoids cultured *in vitro*. **(A)** Hematoxylin and eosin staining and Safranin O-Fast green staining of intervertebral disc cultured *in vitro*. *n* = 6. Scale bar, 100 μm. **(B)** Histologic score of intervertebral disc in each group. **(C)** Immunohistochemical images of COL2 and MMP3 of intervertebral disc. *n* = 6. Scale bar, 100 μm. **(D)** Average percentage of positive staining area of intervertebral disc in each group. Data are presented as the mean ± SD. Significant differences between groups are indicated as ***p < 0.001, **p < 0.01, *p < 0.05.

After observing the differences in the expression of PDGF-BB in the nucleus pulposus of different patients, we would like to further observe the effect of PDGF-BB on intervertebral disc organoids *in vitro*. We separated the lumbar vertebrae of 8-week-old mice by aseptic surgery, and used IL-1β (10 ng/ml) to simulate the organoid model under inflammation. The organoids were cultured with organoid culture medium containing or without imatinib (5 nM) and recombinant PDGF-BB protein (50 ng/ml) for 96 h. Our CCK-8 analysis results suggest that NPCs with imatinib concentration ≤5 nM have no obvious cytotoxicity after 24, 48 and 72 h (Supplementary Figure S1A). H-E staining showed that the intervertebral disc collapsed under the treatment of IL-1β and imatinib, while the SOFG staining showed that the cartilage was lost (Figure 2A). The above-mentioned destructive effect was alleviated after the treatment of PDGF-BB. At the same time, we evaluated the degeneration of the intervertebral disc through the mouse intervertebral disc scoring system proposed by Tam et al. (2018) (Figure 2B). It is worth noting that the immunohistochemical staining results showed that under the action of IL-1β, the expression of ECM anabolic marker COL2 decreased, while the expression of catabolic marker MMP3 increased (Figures 2C,D). After PDGF-BB receptor blockade, ECM catabolism is more obvious, and the difference is statistically significant. However, the recombinant PDGF-BB protein reversed the trend of COL2 and MMP3 induced by IL-1β. This suggests that blocking the PDGF-BB receptor will accelerate the loss of ECM and accelerate the degeneration of the intervertebral disc.

### Platelet-Derived Growth Factor-BB Alleviates Cell Cycle Arrest and Apoptosis in NP Cells

**FIGURE 3:**
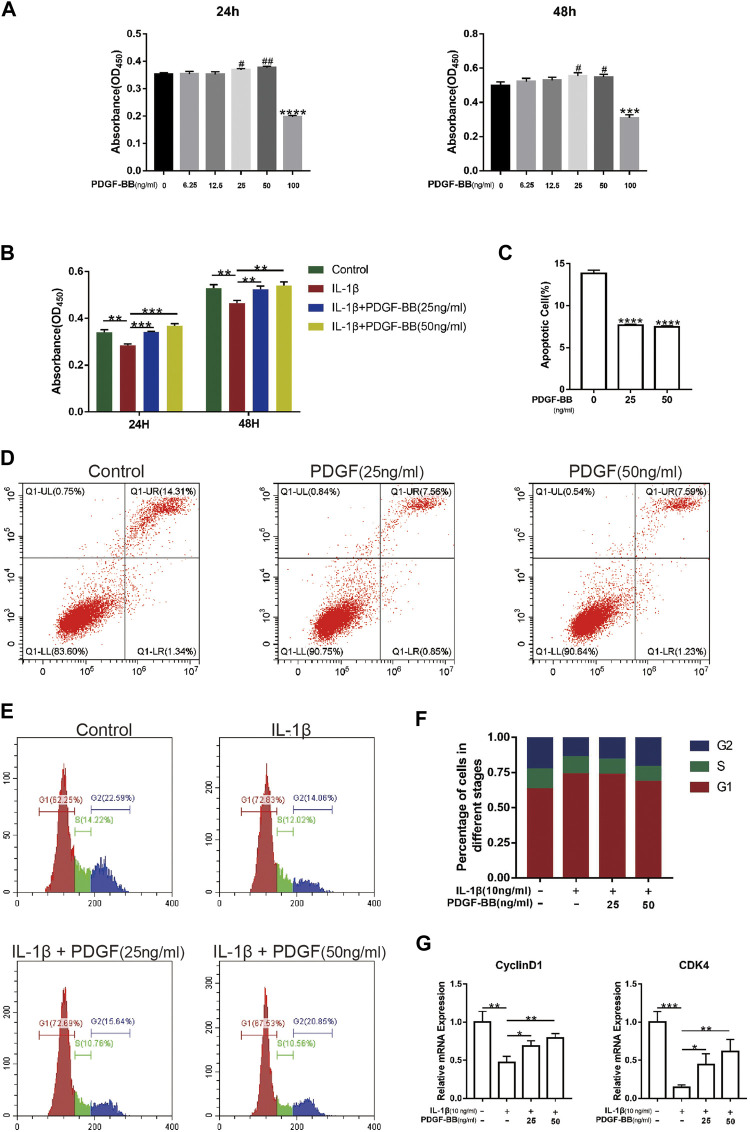
PDGF-BB alleviates cell cycle arrest and apoptosis in NP cells. **(A)** The CCK-8 assay measured effects of the indicated concentrations of the recombinant PDGF-BB protein on the toxicity of NP cells at 24 and 48 h. **(B)** The CCK-8 assay measured effects of PDGF-BB and IL-1β on the cytotoxicity of NP cells at 24 and 48 h. The 96-well plates contained 2,000 NP cells per well. **(C,D)** The apoptosis of NP cells was evaluated by flow cytometry. **(E,F)** The cell cycle of NP cells was evaluated by flow cytometry. **(G)** The mRNA expression levels of CyclinD1 and CDK4 were measured by qPCR. The expression of these genes was normalized to the expression of GAPDH. Data are presented as the mean ± SD. Significant differences between groups are indicated as ****p < 0.0001, ***p < 0.001, **p < 0.01, *p < 0.05.

After determining the protective effect of PDGF-BB on intervertebral discs *in vitro*, we investigated whether PDGF-BB affects the apoptosis and cell cycle of NP cells. Our CCK-8 test results show that the recombinant PDGF-BB protein has no obvious cytotoxicity when the concentration is lower than 50 ng/ml (Figure 3A). So we treated NP cells with IL-1β (10 ng/ml) and recombinant PDGF-BB protein (25 ng/ml, 50 ng/ml) for 24 or 48 h (Figure 3B). It is easy to find that PDGF-BB rescued the decrease in cell viability caused by IL-1β. Flow cytometry showed that the recombinant PDGF-BB protein can effectively inhibit the apoptosis of NP cells (Figures 3C,D). IL-1β inhibits NP cells from entering S phase from G1 phase, but PDGF-BB can promote cell proliferation (Figures 3E,F). The qPCR results also showed that PDGF-BB promoted the expression of CyclinD1 and CDK4, which is related to cell cycle regulation (Figure 3G).

### The Effect of Platelet-Derived Growth Factor-BB on the Anabolism and Catabolism of Extracellular Matrix in Nucleus Pulposus Cells Treated With IL-1β

**FIGURE 4:**
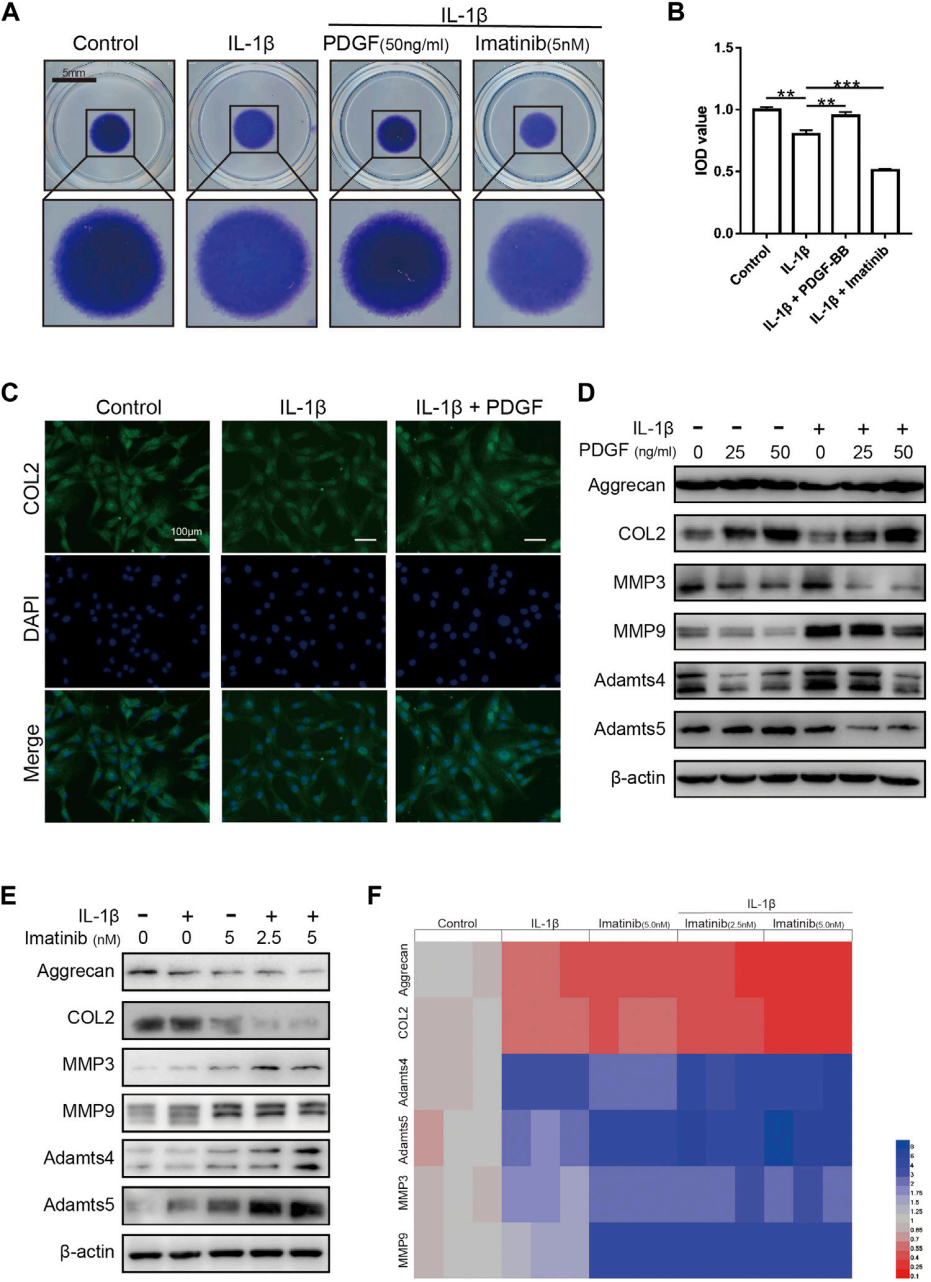
The effect of PDGF-BB on the anabolism and catabolism of ECM in NPCs treated with IL-1β. **(A,B)** Toluidine blue staining of NP cells on high-density culture after treatment with IL-1β, PDGF-BB and Imatinib for 7 days. Scale bar, 5 mm. **(C)** Representative images of NP cells treated with IL-1β and PDGF-BB for 48 h. Scale bar, 100 μm. **(D)** The expression of anabolic-related proteins and catabolism-related proteins were measured by western blot. All NP cells were treated with or without IL-1β (10 ng/ml) and PDGF-BB for 48 h. **(E)** The expression of anabolic-related proteins and catabolism-related proteins were measured by western blot. All NP cells were treated with or without IL-1β (10 ng/ml) and Imatinib for 48 h. **(F)** The mRNA expression levels of Aggrecan, COL2, MMP3, MMP9, Adamts4 and Adamts5 were measured by qPCR. The expression of these genes was normalized to the expression of GAPDH. Data are presented as the mean ± SD. *n* = 3. Significant differences between groups are indicated as ***p < 0.001, **p < 0.01.

The results of high-density cell culture showed that the ECM of NPC incubated with IL-1β was reduced significantly. After blocking PDGF-BB receptor with imatinib, the above-mentioned destructive effect becomes more serious (Figures 4A,B). However, the recombinant PDGF-BB protein reversed the above trend and showed a strong ability to promote ECM synthesis. The results of immunofluorescence assay suggested that PDGF-BB reversed the decrease in COL2 induced by IL-1β, which is one of the main components of ECM (Figure 4C).

Recombinant PDGF-BB protein reversed the trend of ECM anabolic markers aggrecan and COL2, which decreased with IL-1β incubation. At the same time, the levels of ECM catabolism markers ADAMTS-4, ADAMTS-5, MMP3 and MMP9 were inhibited, which increased with the incubation with IL-1β (Figure 4D; Supplementary Figure S1C). It is worth noting that, after the use of PDGF-BB inhibitors, western blot analysis showed that ECM anabolism is further suppressed (Figure 4E; Supplementary Figure S1B). On the basis of the above results, we used PCR to analyze the effect of PDGF-BB inhibitors on the expression of genes related to ECM anabolic and catabolism. PCR results showed that after blocking the PDGF-BB receptor, the expression of anabolism-related genes in ECM decreased, while the expression of catabolism-related genes increased (Figure 4F).

### Effects of Platelet-Derived Growth Factor-BB on the MAPK and PI3K/AKT Pathway in IL-1β-Treated Nucleus Pulposus Cells

**FIGURE 5:**
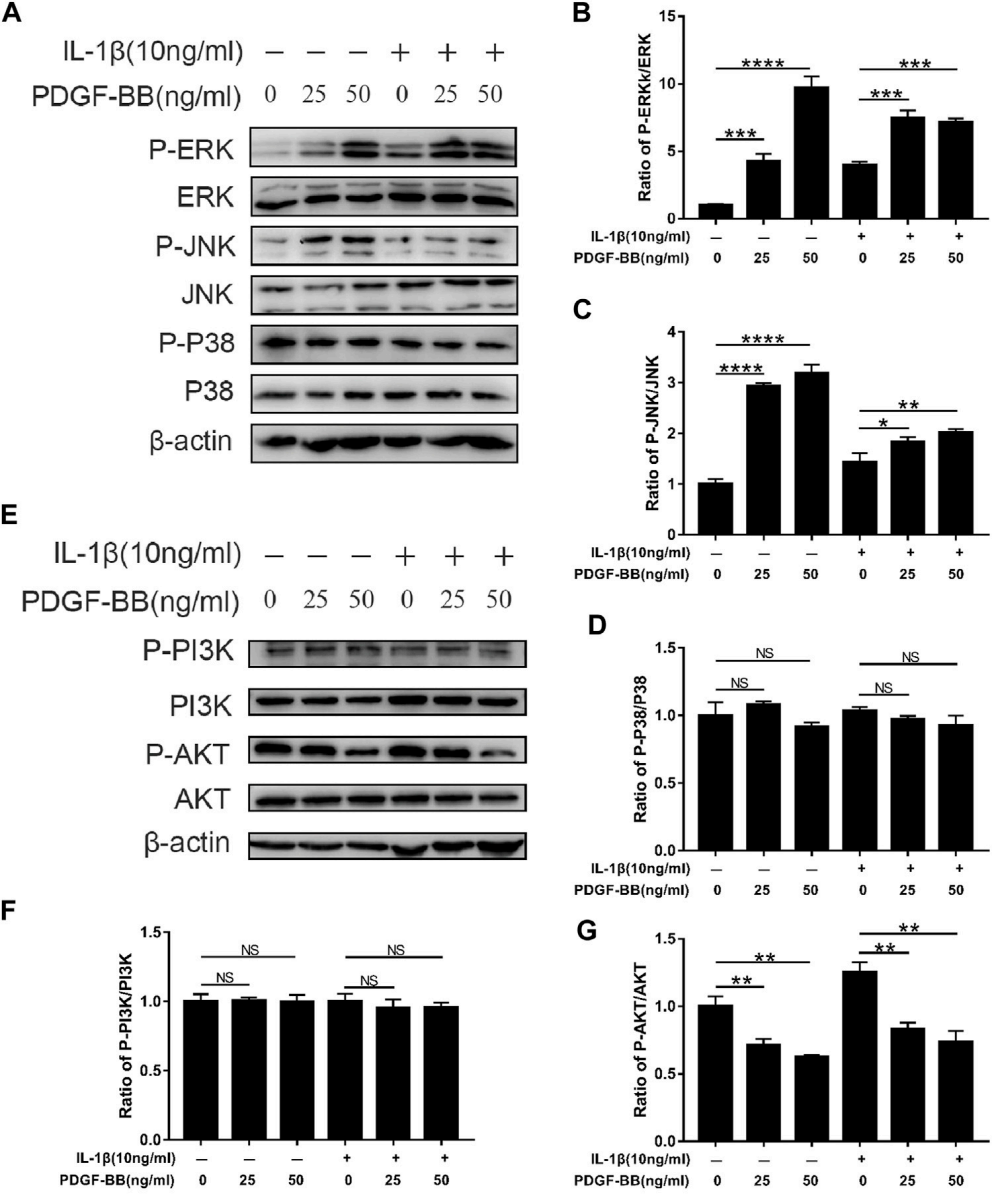
Effects of PDGF-BB on the MAPK and PI3k/AKT pathway in IL-1β-treated NPCs. **(A–D)** Western blot showed the PDGF-BB promotion effects on the phosphorylation of ERK and JNK, but not P38. **(E–G)** Western blot showed the PDGF-BB alleviation effects on the phosphorylation of AKT. All NP cells were treated with or without IL-1β (10 ng/ml) and PDGF-BB for 48 h. The results were quantified using ImageJ software. Data are presented as the mean ± SD. *n* = 3. Significant differences between groups are indicated as ****p < 0.0001, ***p < 0.001, **p < 0.01, *p < 0.05.

In order to explore the mechanism of PDGF-BB regulating NPCs, we studied the effect of PDGF-BB on the MAPK signaling pathway and PI3K/AKT signaling pathway through western blot analysis. Interestingly, the results of western blot analysis showed that the ratio of p-JNK/JNK and p-ERK/ERK was higher in NPCs treated with recombinant PDGF-BB protein, while the ratio of p-P38/P38 did not change significantly (Figures 5A–D). This indicates that PDGF-BB regulates the physiological functions of nucleus pulposus cells by activating the JNK pathway and the ERK pathway, but not the P38 pathway.

In addition, the PI3K of NPCs treated with recombinant PDGF-BB protein did not change significantly. But its downstream signal AKT phosphorylation is significantly inhibited (Figures 5E–G). This suggests that the PI3K/AKT pathway is also involved in the regulation of PDGF-BB on the intervertebral disc.

### Effects of Platelet-Derived Growth Factor-BB on the Pyroptosis Pathway in IL-1β-Treated Nucleus Pulposus Cells

**FIGURE 6:**
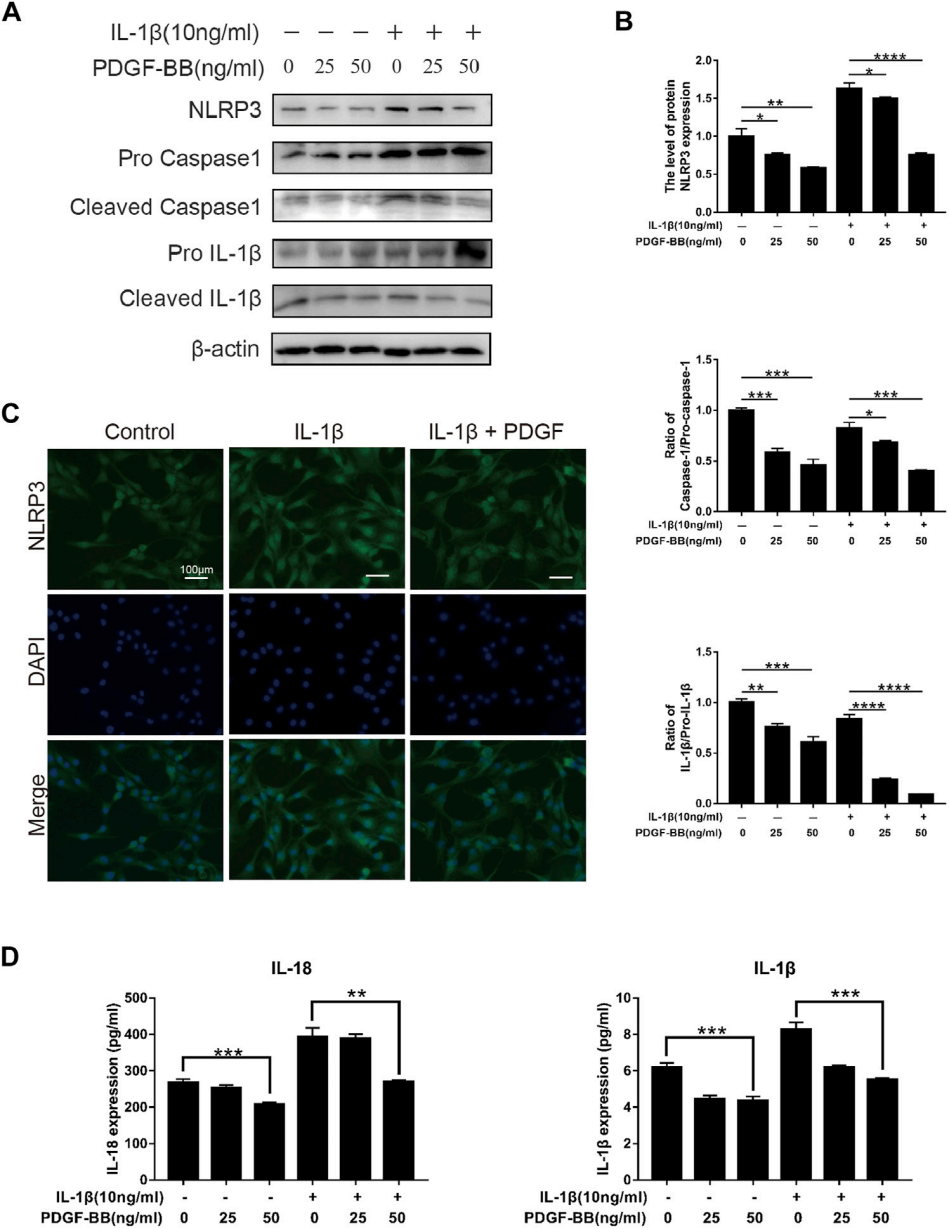
Effects of PDGF-BB on the Pyroptosis pathway in IL-1β-treated NPCs. **(A,B)** Western blot showed that PDGF-BB inhibits the expression of NLRP3 and reduces the ratios of cleaved IL-1β to pro-IL-1β, and cleaved caspase-1 to pro-caspase-1. All NP cells were treated with or without IL-1β (10 ng/ml) and PDGF-BB for 48 h. The results were quantified using ImageJ software. **(C)** Representative images of NP cells treated with IL-1β and PDGF-BB for 48 h. Scale bar, 100 μm. **(D)** ELISA showed that PDGF-BB inhibits the expression of IL-1β and IL-18. Data are presented as the mean ± D. *n* = 3. Significant differences between groups are indicated as ****p < 0.0001, ***p < 0.001, **p < 0.01, *p < 0.05.

In order to explore the effect of PDGF-BB on the pyroptosis of NPCs in an inflammatory environment, we conducted further studies by Western blot and ELISA. Compared with the control group, the expression of NLRP3 and the ratios of cleaved IL-1β to pro-IL-1β, cleaved caspase-1 to pro-caspase-1 were significantly higher in IL-1β-treated NPCs (Figures 6A,B). This means that the pyroptosis pathway is activated in NPCs treated with IL-1β. Compared with IL-1β-treated nucleus pulposus cells, PDGF-BB-treated nucleus pulposus cells showed a significantly lower ratio. This indicates that PDGF-BB can inhibit the scorch death of NPCs in an inflammatory environment. In addition, the results of immunofluorescence assay suggested that PDGF-BB reversed the increase in NLRP3 induced by IL-1β (Figure 6C).

In addition, ELISA results showed that PDGF-BB can significantly inhibit the expression of pyroptosis products IL-1β and IL-18, which can accelerate degeneration of the intervertebral disc (Figure 6D).

### Blocking the Platelet-Derived Growth Factor-BB Receptor Accelerates Disc Degeneration *In Vivo*

**FIGURE 7:**
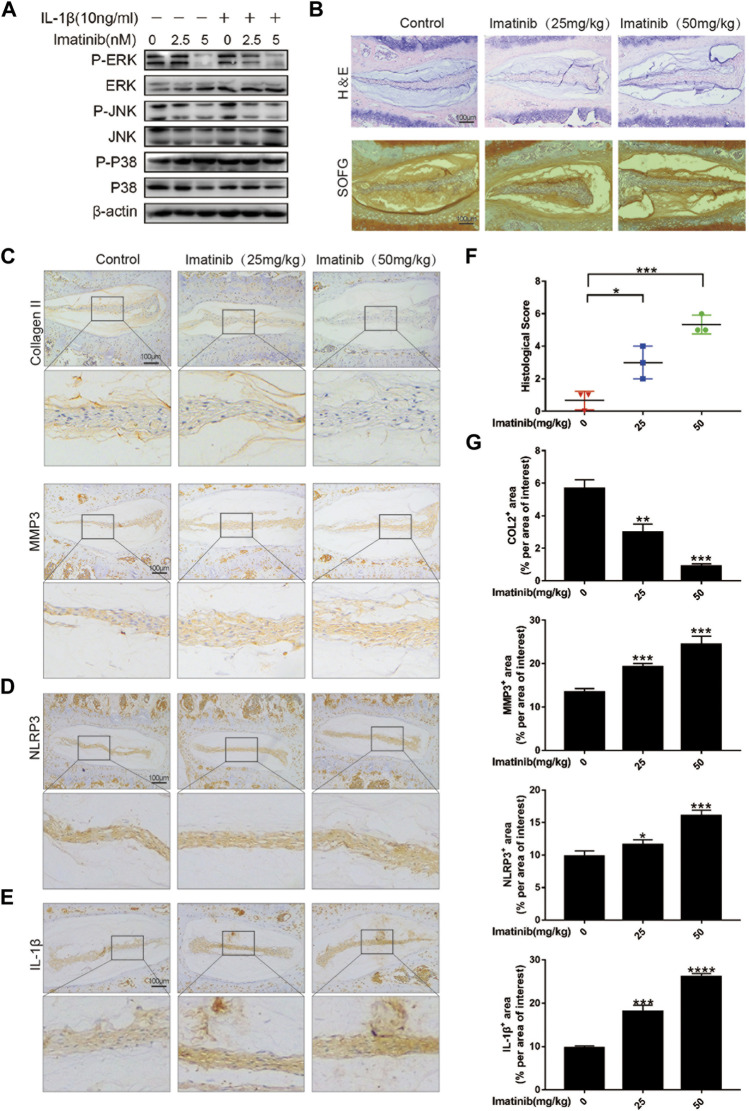
Blocking the PDGF-BB receptor accelerates disc degeneration *in vivo*. **(A)** Western blot showed the Imatinib alleviation effects on the phosphorylation of ERK and JNK, but not P38. **(B)** Hematoxylin and eosin staining and Safranin O-Fast green staining were performed after 8 weeks of intraperitoneal injection. Scale bar, 100 μm. **(C–E)** Immunohistochemical images of COL2, MMP3, NLRP3 and IL-1β of intervertebral disc. *n* = 3. Scale bar, 100 μm. **(F)** Histologic score of intervertebral discs in each group. **(G)** Average percentage of positive staining area of intervertebral disc in each group. Data are presented as the mean ± SD. Significant differences between groups are indicated as ****p < 0.0001, ***p < 0.001, **p < 0.01, *p < 0.05.

Our previous work results showed that after blocking the PDGF-BB receptor, the phosphorylation of ERK and JNK in nucleus pulposus cells was significantly inhibited, but not P38 (Figure 7A; Supplementary Figure S1D). Therefore, in order to verify the role of PDGF-BB *in vivo*, we used 12-week-old mice to intraperitoneally inject imatinib diluent (25 mg/kg, 50 mg/kg) or the same volume of PBS twice a week. Two months later, we extracted the intervertebral disc tissue and prepared paraffin sections. The results of HE staining showed that the IVD fibrosus was disordered under the treatment of imatinib, while the results of SOFG staining showed that the IVD cartilage was lost under the treatment of imatinib (Figure 7B). We performed histological scores on the intervertebral disc in each group, and the results showed that the degree of degeneration increased with the increase of imatinib concentration (Figure 7F).

The results of immunohistochemistry suggested that as the concentration of imatinib increased, the expression of ECM anabolic marker COL2 decreased in turn, while the ECM catabolic marker MMP3 increased in a concentration-dependent manner (Figures 7C,G). In addition, the results showed that imatinib dose-dependently promoted the expression of NLRP3 and IL-1β (Figures 7D,E), and inhibited the expression of P-ERK and P-JNK (Supplementary Figure S1E). These results indicate that blocking the PDGF-BB receptor may accelerate the degeneration of the intervertebral disc by promoting the pyrolysis pathway.

## Discussion

Cytokines, growth factors and enzymes can regulate the internal stability of the intervertebral disc through autocrine and paracrine methods under normal physiological conditions. PDGF-BB, as a growth factor that can stimulate the growth of connective tissue and other tissue cells, has been extensively studied in tumor and angiogenesis. Since Tolonen et al. (1997) first discovered that PDGF-BB is expressed in intervertebral disc, the role of PDGF-BB in the mechanism of intervertebral disc degeneration has received attention (Gruber et al., 2000; Paglia et al., 2016). Our previous work found that PDGF-BB is expressed in nucleus pulposus tissue and NP cells, and there is a difference in the expression of PDGF-BB between the normal disc and the degenerative disc. Therefore, we confirmed that PDGF-BB has a protective effect on intervertebral discs through *in vitro* organoid culture experiments. And further explored the protective mechanism of PDGF-BB, which helps to provide new insights into the pathophysiological mechanism and treatment strategies of intervertebral disc degeneration.

As previous studies have demonstrated, inflammation plays an important role in the process of intervertebral disc degeneration (Sun et al., 2020; Lyu et al., 2021; Wang et al., 2021; Zhang et al., 2021a). Under the action of IL-1β, NPCs lead to the increase of inflammatory factors and the loss of ECM. The ECM produced by the nucleus pulposus cells plays a key role in the stability of the intervertebral disc, and the loss of ECM will further aggravate the degeneration. In this study, we conducted high-density cell culture, Western blot analysis, quantitative PCR and immunofluorescence experiments to confirm the effect of PDGF-BB on the production of ECM by NP cells. It is worth noting that PDGF-BB shows a strong ability to promote ECM anabolism. And PDGF-BB promoted the expression of COL2 and Aggrecan, and inhibited the expression of MMPs, ADAMTS4 and ADAMTS5.

Pyrolysis is a kind of programmed cell death, which is manifested by the continuous expansion of cells until the cell membrane ruptures and release of cell contents. Different from apoptosis, cells that undergo pyrolysis will release cell contents to further activate the strong inflammatory response in the surrounding environment. At present, more and more evidences show that pyrolysis plays an important role in the pathogenesis of degenerative disc disease. Therefore, we explored the effect of PDGF-BB on the pyrolysis of nucleus pulposus cells induced by IL-1β (Bai et al., 2020; Liao et al., 2021; Zhou et al., 2021). We found that PDGF-BB can effectively inhibit the activation of pyroptosis-related signals and the production of pyroptosis products in a concentration-dependent manner. In addition, experiments *in vivo* animal showed that with the increase in the concentration of PDGF-BB receptor blocker imatinib, the degeneration of intervertebral discs in mice became more serious. It is worth noting that the expression of NLRP3 and IL-1β also increased after mice were injected with imatinib intraperitoneally.

At the same time, we also tested the effect of PDGF-BB on the MAPK signaling pathway and the PI3K/AKT signaling pathway. Interestingly, PDGF-BB promoted the phosphorylation of ERK and JNK, but had no significant effect on the phosphorylation of P38. In addition, PDGF-BB also inhibited the phosphorylation of AKT, a downstream signal of PI3K. The above results suggest that PDGF-BB mainly activates the ERK and JNK pathways instead of the P38 pathway, and inhibits the PI3K/AKT signaling pathway to regulate the function of nucleus pulposus cells.

Of course, our experiment still has shortcomings. The MAPK pathway is generally considered to be an inflammation-related pathway, and our experimental results suggest that PDGF-BB promotes the phosphorylation of the MAPK pathway. It has been reported that the increased phosphorylation of the MAPK pathway may be due to feedback activation pathways or crosstalk related to parallel signaling pathways (Pratilas and Solit, 2010). However, we have not conducted a more in-depth study on the MAPK pathway, especially the downstream signaling molecules of ERK and JNK. Therefore, we have no evidence to rule out whether the phosphorylation of ERK and JNK is a feedback activation caused by the inhibition of downstream signals. In addition, as previous studies have shown, PDGF-BB’s regulation of intervertebral discs is multi-targeted, which means that its protective effect may not be mainly through the MAPK pathway. Overall, our study found that PDGF-BB can inhibit IL-1β-induced inflammation and pyroptosis, promote the synthesis of ECM in the intervertebral disc, and protect it from degeneration. And our results indicate that PDGF-BB holds great therapeutic potential to treat IDD.

## Data Availability

The original contributions presented in the study are included in the article/Supplementary Material, further inquiries can be directed to the corresponding authors.
