# Supplementary material for: Platelet-Derived Growth Factor-BB Inhibits Intervertebral Disc Degeneration via Suppressing Pyroptosis and Activating the MAPK Signaling Pathway
Source: Front Pharmacol. 2022 Jan 14;12:799130. doi: 10.3389/fphar.2021.799130 (PMC8795915; doi:10.3389/fphar.2021.799130)
Supplement: Supplementary file 1 [file DataSheet1.docx]

**
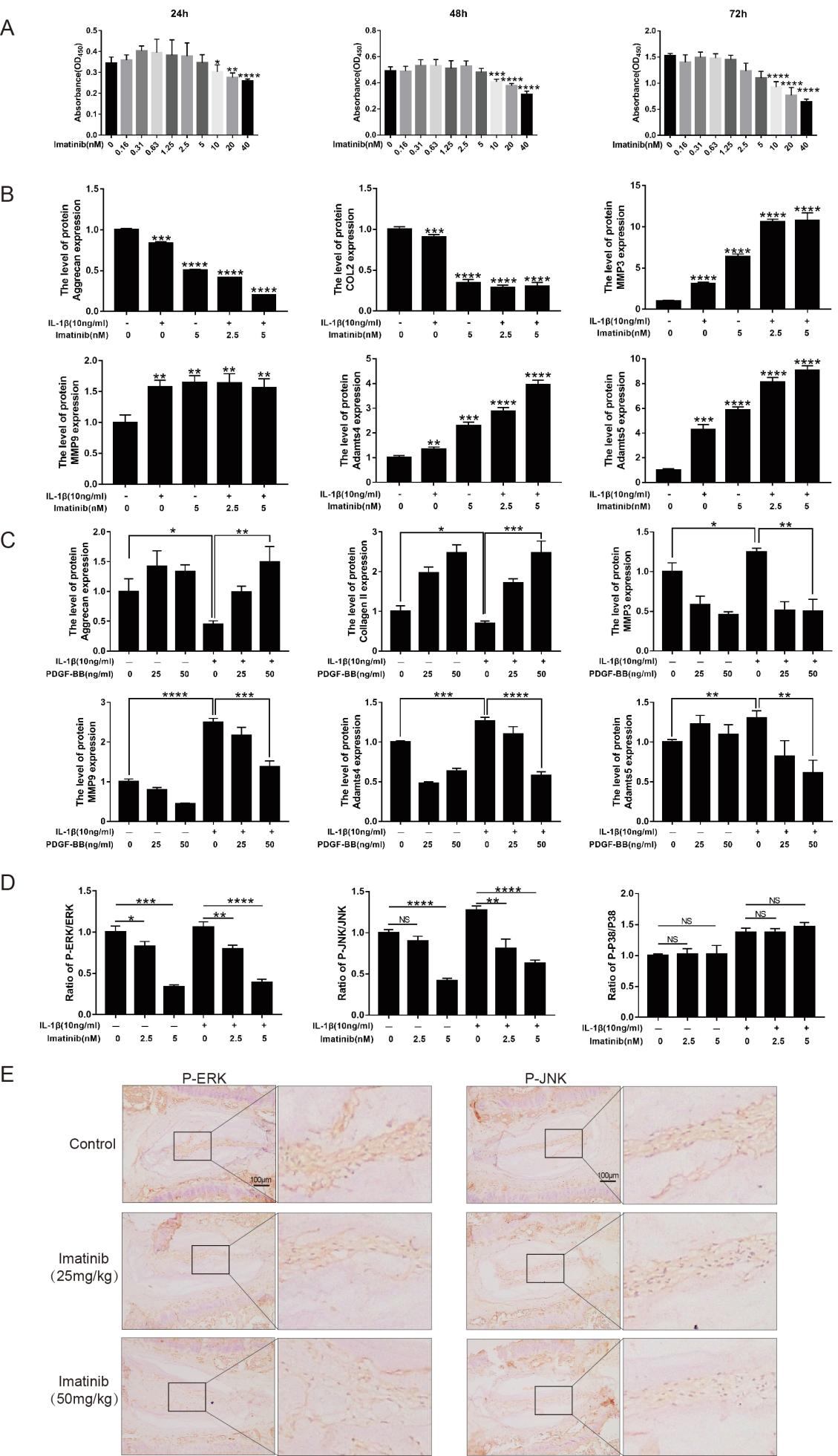
**

**Supplementary Figure 1. (A)** The CCK-8 assay measured effects of the indicated concentrations of imatinib on the toxicity of NP cells at 24, 48 and 72 h. The 96-well plates contained 2,000 NP cells per well. **(B)** The expression of anabolic-related proteins and catabolism-related proteins were measured by western blot. All NP cells were treated with or without IL-1β (10 ng/ml) and imatinib for 48 h. The results were quantified using ImageJ software. **(C)** The expression of anabolic-related proteins and catabolism-related proteins were measured by western blot. All NP cells were treated with or without IL-1β (10 ng/ml) and PDGF-BB for 48 h. The results were quantified using ImageJ software. **(D)** Western blot showed the imatinib inhibits the phosphorylation of ERK and JNK, but not P38. All NP cells were treated with or without IL-1β (10 ng/ml) and imatinib for 48 h. The results were quantified using ImageJ software. n=3. Data are presented as the mean ± S.D. Significant differences between groups are indicated as ****P < 0.0001, ***P < 0.001, **P < 0.01, *P < 0.05. **(E)** Immunohistochemical images of P-ERK and P-JNK of intervertebral disc. n=3. Scale bar, 100μm.
